# The role of exergaming in Parkinson’s disease rehabilitation: a systematic review of the evidence

**DOI:** 10.1186/1743-0003-11-33

**Published:** 2014-03-07

**Authors:** Gillian Barry, Brook Galna, Lynn Rochester

**Affiliations:** 1 Clinical Ageing Research Unit, Institute for Ageing and Health, Campus for Ageing and Vitality, Newcastle University, Newcastle upon Tyne NE4 5PL, UK

**Keywords:** Nintendo Wii, Kinect, Balance, Exercise, Computer games

## Abstract

Evidence for exercise based computer games (exergaming) as a rehabilitation tool for people with Parkinson’s disease (PD) is only now emerging and is yet to be synthesised. To this end, we conducted a systematic review of the exergaming literature to establish what is known about the safety, feasibility and effectiveness of exergaming for rehabilitation of motor symptoms experienced by people with PD. Seven electronic databases were searched for key terms surrounding exergaming and PD. Data were extracted by two reviewers independently. From an initial yield of 1217 articles, seven were included in the review. Six studies used commercial games with the Nintendo Wii fit platform. The scientific quality of reporting was generally good, however the overall methodological design of studies was weak, with only one randomised controlled trial being reported. Safety: Participant safety was not measured in any of the studies. Feasibility: People with PD were able to play exergames, improve their performance of gameplay and enjoyed playing. However, one study observed that people with PD had difficulty with fast and complex games. Effectiveness: Six studies showed that exergaming elicited improvements in a range of clinical balance measures or reduction in the severity of motor symptoms. Results from the only randomised controlled trial showed that exergaming was as effective as traditional balance training for people with PD to improve the UPDRS II, standing balance and cognition, with improvements in both groups retained 60 days after the training ended. In conclusion, exergaming is an emerging tool to help rehabilitate motor skills in people with PD. Although we were able to establish that exergaming is feasible in people with PD, more research is needed to establish its safety and clinical effectiveness, particularly in the home. The use of commercial games may be too difficult for some people with PD and exergames tailored specifically to the rehabilitation needs and capabilities of people with PD are required for optimal efficacy, adherence and safety.

## Introduction

Exercise is emerging as an efficacious therapy to compliment the rehabilitation of problematic motor symptoms in Parkinson's disease (PD) such as gait and balance which contribute to reduced mobility and increased risk of falls [1,2]. Delivery of exercise as part of a rehabilitation programme remains challenging in terms of adherence, acceptability, access and cost. There is growing interest in exergaming as a potential rehabilitation tool to facilitate disease specific exercise in many clinical groups [3-5].

Exergames are computer games that are driven by gross physical movements of the player. They work by combining real-time motion detection with engaging video games that can help motivate people to exercise. Exergaming as a therapeutic tool incorporates functional, purposeful and engaging exercise in a quantifiable and reliable way [6-8]. It has shown benefits in traumatic brain injury [9,10], Cerebral Palsy [11], stroke rehabilitation [12,13], young adults with physical and intellectual disabilities [5], and older adults [14,15]. Emerging evidence in older adults suggests that playing exergames may also improve cognitive function [16]. Exergaming may therefore offer a low cost, home-based tool to augment traditional rehabilitation of motor symptoms in people with PD. Home use and tailored training may also facilitate exercise compliance and motivation [17].

The use of exergaming for PD rehabilitation shows great promise however the evidence has not yet been formally reviewed or synthesised. Critical questions remain surrounding the use of exergaming for people with PD, especially in relation to the prescription of home-based exercise and the suitability of commercial games. To address these questions, we conducted a systematic review of the literature to evaluate the evidence for the safety, feasibility and effectiveness of exergaming as a rehabilitation tool in people with PD.

## Methods

The following databases were searched electronically in January 2013 and updated in December 2013: Web of science (1864-2013), CINAHL (1982-2013), Scopus (1960-2013), Science Direct (1823-2013), IEEE (1872-2013), PubMed (1869-2013) and Cochrane (1949-2013). We searched the titles, keywords and abstracts of database entries using the search strategy: (Exergam* OR active video gaming OR Microsoft Kinect OR Kinect OR Nintendo Wii OR Wii OR Sony EyeToy OR IREX OR Dance Dance Revolution) AND (Parkinson*), where * denotes a wildcard to allow for alternate suffixes. We also searched the grey literature (such as generic internet search engines) to avoid missing relevant articles. Inclusion Criteria: Articles were included if they reported an exergaming based intervention in people with PD and were full scientific papers written in English. Exclusion criteria: Conference posters and abstracts were excluded. Papers that were virtual reality based treadmill interventions were excluded as we focused on interventions that could be practically implemented in the home.

The methodological quality of each article was assessed using a customised quality assessment tool based on previous systematic reviews [18] (see Table 1 for a list of items and Table 2 for the scoring criteria). To ensure valid scoring of the quality assessment, two reviewers (GB and BG) independently scored the quality of the articles. Incongruities between the two reviewers were discussed, with a third reviewer assessing any unresolved differences in extraction.

**Table 1:** Methodological quality assessment tool

**Question number**	**Question**
1.	Are inclusion and exclusion criteria stated?
2.	Are participant characteristics described in detail?
3.	Was sample size justified?
4.	Was randomisation of groups explained?
5.	Was the design clearly stated?
6.	Were exergaming sessions explained in detail?
7.	Were baseline and post testing data presented?

**Table 2:** Methodological quality assessment scores of each study

**Question**	**Scoring criteria**	**Assad et al.**[25]	**Zettergren et al.**[22]	**Pompeu et al.**[21]	**Mendes et al.**[20]	**Esculier et al.**[19]	**Mhatre et al.**[23]	**Hertz et al.**[24]
Inclusion/exclusion criteria detailed	1 = yes; .5 = yes lacking detail; 0 = no	0	1	1	1	.5	1	1
Participant characteristics detailed	Number	1	1	1	1	1	1	1
	Age	1	1	1	1	1	1	1
	Sex	1	1	0	0	1	1	1
	Disease severity	.5	0	1	1	1	1	1
Sample size justified	1 = yes; 0 = no	0	0	1	1	0	1	0
Randomisation explained	1 = yes; .5 = yes lacking detail; 0 = no	na	na	1	na	na	na	na
Research design clearly stated	1 = yes; .5 = yes lacking detail; 0 = no	.5	1	1	1	1	1	0
Exergaming sessions explained	1 = yes; .5 = yes lacking detail; 0 = no	.5	1	1	1	1	1	1
Baseline and post test data presented	1 = yes; .5 = yes lacking detail; 0 = no	.5	1	1	1	1	1	1

na = indicates not applicable.

Data relating to the safety, feasibility and effectiveness were extracted from each study. For the purposes of this review: *Safety* referred to any subjective (researcher, clinician or participants perspectives) or objective measures (fall or near falls); *Feasibility* referred to whether people were able to play the games, whether they improved in their gameplay and whether they enjoyed and felt motivated by the gameplay; and *Effectiveness* referred to whether participants improved on clinical tests of motor performance (including balance) or disease severity, and whether these improvements were retained after the exergaming intervention. One reviewer (GB) screened the initial 1121 titles and abstracts before the full text of 10 publications was screened by two reviewers (GB & BG). Four publications were excluded as they did not include an exercise intervention, rather, they assessed the psychometric properties of the Wii balance board and Wii remote for people with PD.

## Results

**Figure 1:**
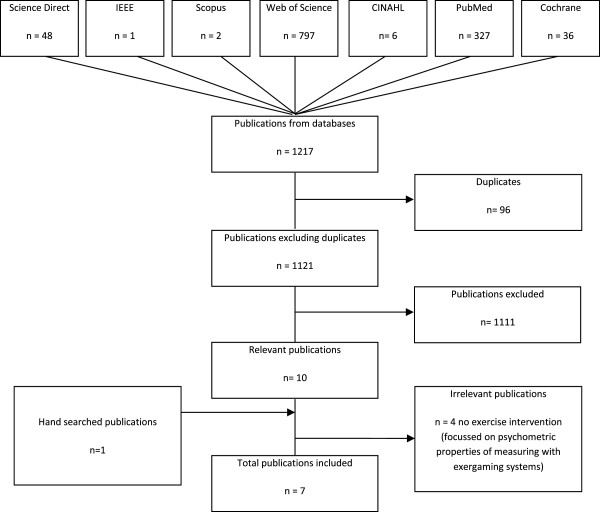
Results of the literature search strategy conducted in December 2013.

Our search yielded 1121 articles (excluding duplicates, Figure 1) relating to exergaming and Parkinson's disease (PD). Six papers from the original search and one article found in the grey literature were included in the review.

### Study design and methodological quality

Table 2 summarises the methodological quality of each study. Adequate details were provided to replicate the studies. The aims, inclusion and exclusion criteria, and exergaming interventions were well described. Six studies used commercial Wii games [19-24] and one developed a game designed for PD using the Sony Playstation Eye™ camera [25]. Participant demographic characteristics and the severity of their PD were generally well reported although the use of several different scales of disease severity (Hoehn and Yahr [26], UPDRS [27] and disease duration) made comparisons between studies difficult and may limit future meta-analyses. The rationale for sample size was provided in three studies.

Table 3 summarises the study design, exergaming system and intervention. The studies included two longitudinal trials (with healthy control groups) [19,20], a participatory design study [25], a case study [22] a randomised controlled trial [21], a prospective interventional cohort study [23] and a pre-post test design [24]. Six of these studies trained people with PD over 12-18 sessions (over 4-8 weeks), and measured performance on clinical tests directly before and after the intervention [19-24]. A further three studies tested whether improvements in clinical tests were retained 60 days after the intervention [20,21,24]. The final study outlined a game designed specifically for people with PD using the Sony Playstation Eye™ and tested its feasibility during one session in a group of 13 people with PD [25].

**Table 3:** Summary of participant characteristics, study design and exergaming interventions

**Author**	**Participants**	**PD diagnosis**	**Design and aim**	**System**	**Games**	**Intervention**	**Setting**
**Assad et al. [**[25]**]**	13 PD (Aged 54-86 years) 9 males and 5 females	Mean duration of PD = 9 yr	**Participatory design**	Sony Playstation Eye™	5 Upper body movement games.	1 session Duration NS	Clinic
			Assess PD subjects’ perception of playing an exergame developed for PD.				
**Zettergren et al. [**[22]**]**	1 male (69 years old)	PD duration <3 yr	**Single subject case study**	Nintendo Wii™	Penguin slide, Table tilt, Balance bubble, Free step, Island cycling, Obstacle course and Rhythm parade.	2 sessions/week 8 weeks 40-60 minutes per session	Clinic
			Evaluate the effects of multiple Nintendo Wii Fit activities on gait, balance and mobility.				
**Pompeu et al. [**[21]**]**	32 PD (Aged 60-85 years)	HY I & II	**Single-blind randomized controlled trial (RCT)**	Nintendo Wii™	Balance games: (Table tilt, Tilt city, Penguin slide, Soccer heading) Static balance (Torso twist and Single leg extension).	2 sessions/week 7 weeks 60 minutes per session	Clinic
					Stationary gait: (Rhythm parade, Obstacle Course, Basic Step and Basic Run)		
			Test if PD patients improve their performance on the Wii & compare the effects of Wii exercise on motor and cognitive training with balance therapy.				
**Mendes et al. [**[20]**]**	16 PD (No mean age data) 11 Healthy elderly	HY I = 2; HY II = 14	**Longitudinal controlled trial**	Nintendo Wii™	Table tilt, Tilt city, Penguin slide, Soccer heading, Obstacle course, Rhythm parade, Basic run plus, Basic step, Single leg extensions games.	2 sessions/week 7 weeks 60 minutes per session	Clinic
			To evaluate the learning, retention and transfer of performance after using the Wii.				
**Esculier et al. [**[19]**]**	10 PD (61.9 ± 11.0)	Mean duration of PD = 8.5 (3.6) years	**Longitudinal controlled trial**	Nintendo Wii™	Wii sports: Table tilt, Ski slalom, Balance bubble, Ski jump and Penguin slide.	3 × 6 weeks 40 minutes each session	Home
	8 Healthy elderly (63.5 ± 12.0)		To evaluate whether PD subjects balance and functional activities improved using the Wii Compare the effects against a healthy elderly sample.				
**Mhatre et al. [**[23]**]**	10 PD (Aged 44-91 years) 6 female and 4 males	Mean Duration of PD = 6.7 years	**Prospective interventional cohort study**	Nintendo Wii™	Balance board games: Marble game, Skiing, and bubble game.	3 sessions/week 8 weeks 30 minutes each session	Clinic
			Assess the effectiveness of using the Wii Fit on people with PD for gait and balance training.				
**Hertz et al. [**[24]**]**	20 PD (66.7 ± 7.2) 13 male and 7 female	Mean Duration of PD = 5.5 (4.3) years	**Pre-post design trial**	Nintendo Wii™	Tennis, boxing and bowling	3 sessions/week 4 weeks 60 minutes each session	Clinic
			Assess the effectiveness of Wi on both motor and non–motor symptoms of PD.				

HY = Hoehn and Yahr stage.

### Sample Characteristics

The number of people with PD in each study ranged between 1 and 32 (see Table 3). A broad age range was represented across studies with participants ranging from 44-91 years, and both males and females were included. People with PD had relatively mild symptoms (Hoehn & Yahr stage I & II) and were tested whilst on their medication, although medication status was not reported by Assad et al. [25].

### Safety

Table 4 summarises the safety, feasibility and effectiveness of the interventions. Only two studies addressed patient safety, whereby the researchers set up a Wii platform in the participants home and monitored the first exercise session to ensure their safety [19]. Otherwise, neither objective (such as falls or near falls) nor subjective (participant perceptions) measures of safety were reported in any of the studies.

**Table 4:** Safety, feasibility and effectiveness of exergaming interventions

**Author**	**Safety**	**Feasibility**			**Effectiveness**	
		**Gameplay (perception)**	**Game play**		**Clinical Test**	
			**Improvement post intervention**	**Retention**	**Improvement post intervention**	**Retention**
**Assad et al.**[25]	n/s	5/13 participants reported having success during the game and this was the main reason for having fun. 3/13 subjects criticized the game for not having clear goals. Participants like the fairy tale theme to the game and most would play the game with their children or grandchildren.	n/s	n/s	n/s	n/s
**Zettergren et al.**[22]	n/s	n/s	Sun Salutation, Half Moon, Chair, Rowing, Squats, Torso Twist, Penguin Slide, Table Tilt, Balance Bubble, Free Step.	n/s	Berg Balance Scale, Timed up and Go, Gait Speed.	n/s
**Pompeu et al.**[21]	n/s	n/s	Static balance (Single leg extension and Torso Twist), Dynamic balance (Table Tilt, Tilt City, Soccer Heading, and Penguin Slide), Stationary gait (Rhythm Parade, Obstacle Course, Basic Step and Basic Run).	Improvements retained	UPDRS-II, Berg Balance Scale, Unipedal stance eyes open, Unipedal stance eyes closed, Montreal Cognitive, Assessment.	Improvements retained
**Mendes et al.**[20]	n/s	n/s	Similar learning curve for 7 Wii fit games in PD compared to controls (Table tilt, Rhythm parade, Tilt city, Single leg extension, Basic step, Torso twist, Penguin slide), yet did not learn the fast and complex games as well as the controls (Obstacle course, Basic run plus, Soccer heading).	Improvements retained	Functional reach test.	Improvements retained
**Esculier et al.**[19]	First home session supervised by research staff to ensure safe and effective training.	55% of liked the games very much, 33% liked it and 17% were neutral, and no subject disliked playing the Wii. Favourite games included; Ski Jump, Ski Slalom and Table Tilt.	n/s	n/s	Timed up and go, Sit to Stand, Unipedal and bipedal standing balance, 10 m walking speed, Community Balance and Mobility Assessment (CBM), Tinetti Performance Orientated Mobility Assessment (POMA).	n/s
**Mhatre et al.**[23]	Exercise sessions were supervised and a balance bar was available if needed during gaming	n/s	n/s	n/s	Berg Balance Scale, Dynamic gait index, postural sway (eyes open static and dynamic).	n/s
**Hertz et al.**[24]	n/s	n/s	n/s	n/s	Nottingham Extended Activities of Daily Living Test (NEADL) post intervention, PDQ decrease in ADL, emotion, communication, bodily discomfort.	NEADL decreased post intervention. PDQ mobility, ADL and emotion remained improved at 4 weeks post intervention.
						UPDRS motor scores decreased from baseline to post intervention and remained decreased at 4 weeks post intervention.
					UPDRS motor scores, timed tapping test (right side only), Purdue score (left side only), 9-hole peg test (right side only), and time up and go (TUG).	

n/s = not stated; UPDRS = Unified Parkinson’s Disease Rating Scale.

### Feasibility

Three studies measured performance in gameplay before and after an exergaming intervention (14 sessions [20,21] or 16 sessions [22]). In all three studies, people with PD improved their performance on the games. Two of these studies tested participants 60 days after the cessation of the intervention and reported that improvements in gameplay were retained [20,21]. However, Mendes et al. also found that people with PD failed to improve in two games that required fast reactions in response to virtual targets and did not learn as quickly as controls in those games that required dual tasking [20].

Only one study offered a detailed review of the game playing experience [25]. Good levels of motivation during game play were reported although difficulties with the fast pace and cognitive complexity of some games were raised. Esculier et al., presented the only study to use home-based exergaming and used participant logbooks to assess satisfaction of the exergaming intervention [19]. Fifty percent liked the exercise program very much, 33% liked the program, and 17% rated the program as neutral, with no subjects disliking the program.

### Effectiveness

The only randomised controlled trial included in our review tested the effects of playing commerical games on the Nintendo Wii for 14 sessions (over 7 weeks) compared to traditional balance training in 32 people with mild PD (16 in each group) [21]. The primary outcome was section II of the Unified Parkinson’s Disease Rating Scale (UPDRS) and secondary outcomes included balance and cognitive tests. Both groups also received stretching, strengthening and axial mobility exercises. There were no differences between the two training groups at baseline and both groups improved by the same amount after 7 weeks on the primary or secondary measures. When data from both groups were combined, small improvements were observed in the UPDRS II, Berg balance scale, single leg stance (eyes open and eyes closed, but not eyes open with a dual task) and Montreal Cognitive Assessment (MoCA) immediately after training. These improvements were retained 60 days after the final training session.

Five further studies reported improvements on clinical tests in response to an exergaming intervention using commercial Nintendo Wii balance board (Wii Fit) games [19,20,22-24]. However, none of these studies included a control intervention. First, Esculier et al. showed that 18 sessions (over 6 weeks) of exergaming resulted in people with PD being able to stand on one leg for longer (with eyes open but not closed), improved bipedal standing balance with their eyes open (but not closed), and faster performance on the timed up and go, sit to stand and 10 m walk tests [19]. Participants also scored better on the Community Balance and Mobility scale after the intervention. Second, Mendes et al. found 14 sessions of exergaming elicited improvements in the functional reach test immediately after the last training sessions, which was retained 60 days after the last session [20]. Zettergren et al. showed that the gait speed, timed up and go, and Berg balance scale all improved in a case study of one 69 year old man with PD [22]. Mhatre et al. found significant improvements in Berg Balance Scale, Dynamic Gait Index and static balance over an 8 week intervention, [23]. Hertz found significant improvements in in the Nottingham Extended Activities of Daily Living Test (NEADL) at post intervention (12 sessions), however these improvements were short lived as the NEADL scores returned to baseline levels four weeks post intervention. Quality of life as assessed by the PDQ-39 showed significant improvement post intervention for ADL, emotion, communication, bodily discomfort and total score. At 4 week post intervention only ADL and emotion remained significantly improved with the addition of mobility significantly improving. The UPDRS motor score showed a significant decrease post exercise and improvements were retained 4 weeks post intervention. Other outcome measures which showed significant improvement post intervention were the timed up and go (TUG), timed tap (right side, Purdue score (left-side) and 9 – hole peg test score (right side).

## Discussion

The aim of this systematic review was to explore whether exergaming is a safe, feasible and effective rehabilitation tool for people with PD. Exergaming as a method of rehabilitation for people with PD is still very novel, as seen by the small number of studies included in this review. These preliminary studies indicate that exergaming is feasible for PD however games may need to be tailored towards specific clinical populations, and safety and feasibility as a home-based rehabilitation tool is yet to be fully established.

### Safety

More evidence is needed regarding the safety of people with PD playing exergames before it can be recommended for wide spread clinical use, particularly in home-based settings. For this reason, we recommend future studies report both objective and subjective measures of safety. Importantly, six of the seven studies we reviewed used the Nintendo Wii and Wii fit balance board [19-24]. The Wii fit is a raised platform and so may present a trip risk for people with PD, particularly when they are focused on the television screen. New commercial exergaming systems are now available, such as the XBOX Kinect™, that do not require a raised platform. Using these systems may improve the safety of exergaming for people with PD, however this remains to be tested, as does the clinical efficacy of these new exergaming systems.

### Feasibility

Three studies reported improvement in gameplay performance in PD participants using commercial Nintendo Wii exergames [20-24], suggesting commercial games are feasible. Two studies that sought participant feedback about gameplay and reported that people with PD enjoyed playing the games and were motivated to play them [19,25]. Despite these promising results and the potential of commercial exergames as a means of low-cost home-based exercise, there is concern that the commercial games are too difficult for some people with PD [28]. This reflects the need for appropriate game selection and design. A key advantage of exergames is that they can provide immediate biofeedback of performance. This is a useful attribute that may be exploited to improve motor learning in people with PD, especially given their increased reliance on visual cues. Setting an appropriate threshold of difficulty is essential. If feedback is too negative, for example when playing games that are too fast or complex, motivation, adherence and safety may suffer. This was particularly highlighted when Mendes et al. assessed motor and cognitive ability of game play using the Nintendo Wii for PD and healthy elderly adults [20], and showed that PD participants failed to improve on games that required fast decision making and movements to avoid virtual obstacles.

Of the reviewed papers, Assad and colleagues were the only authors to design their own exergame specifically tailored towards people with PD (WuppDi) [25]. Five games were developed to rehabilitate upper body movements and elements of cognition. Games were played, either with a hand held marker (wooden stick) or with no markers. The results showed that the majority of the participants enjoyed the experience and gained positive feedback from playing the games, and would enjoy playing with others especially their grandchildren. However, the authors noted some of the games were too complex and needed to be made easier for people with PD. Some participants also had difficulty with the hand held controllers. Hand held devoices may not be appropriate for people with severe tremor or dyskinesia who could find it difficult to keep a hold of and manipulate the controller.

### Effectiveness

There is also little known regarding the efficacy of exergaming as compared to traditional rehabilitation. Pompeu et al. has conducted the only randomised controlled trial of exergaming for people with PD [21]. The findings of the study suggest that 14 sessions (over 7 weeks) of playing the Nintendo Wii™ can improve clinical measures of balance to the same extent as traditional balance training in people with PD. A potential confounder to this study was that participants in both groups also received stretching, strengthening and axial mobility exercises before each session. As such, it is difficult to conclude whether changes in the clinical tests can be ascribed to the balance training (either traditional or exergaming). Bateni found similar results when analysing the effects of the Wii on balance in healthy elderly adults, in that a combination of the Wii and standard balance training had the greatest improvement in balance outcomes at post test compared to Wii or balance training alone [29]. Therefore, it is possible that exergaming may be more useful for PD as an adjunct to standard clinical treatment than as a stand alone intervention.

Six of the seven studies examined whether people with PD improved on clinical tests after an exergaming intervention [19-24]. All six showed that people with PD improved on various clinical measures of balance (Berg balance score, single leg stand, functional reach test), motor function (Sit to stand, Timed up and go, 10 m walk, timed tapping) and severity of PD motor symptoms (UPDRS II). Two of these studies also showed that improvements in clinical tests were retained 60 days after the intervention [20,21]. With Hertz et al. showing improvements were attained 4 weeks post intervention for PDQ-39 ADL and emotion, and motor scores for UPDRS [24]. These results indicate that exergaming may be effective for the rehabilitation of motor and balance symptoms in people with PD. However, more rigorous research designs need to be adopted in future trials to confirm whether improvements in these tests are clinically meaningful and are not due to increased familiarity with the clinical tests.

One of the potential benefits of exergaming interventions is that they can be administered in the home [17]. Although not formally tested, the resources required for exergaming are likely to be less than those required by formal rehabilitation services. To date, there has only been one home-based exergaming intervention reported in people with PD [19]. The authors were able to show that people with PD improved on several clinical tests and on gameplay over the 6 week training period. Questions remain, however, whether home-based exergaming interventions are safe and as effective as traditional clinic based interventions for people with PD.

### Limitations

We included only full articles in this review, which resulted in the exclusion of two relevant abstracts relating to exergaming and PD [30,31]. Dos Santos et al., showed that 14 sessions of dance based exergaming using a balance board resulted in improved balance and reduced motor symptoms (UPDRS) in people with PD [30]. Alvarez et al. trained 12 people with PD using three commercial XBOX Kinect™ games. Twenty-four sessions over eight weeks resulted in improvements in the UPDRS (II&III) and Tinetti scale for gait and balance [31]. Another limitation of this review was the heterogeneity of the different exergames included in each intervention limited our ability to distinguish which particular games, and which aspects of those games, are useful for rehabilitation of specific symptoms in PD.

### Recommendations

This systematic review indicates that exergaming appears feasible for people with PD, however it also highlights the current paucity of evidence for exergaming as a rehabilitation tool. The studies we reviewed adopted relatively weak designs and small sample sizes. As such, we can not state with confidence whether or not exergaming is a safe and effective rehabilitation tool until larger randomised controlled trails are conducted, especially in a home-based setting. In addition, exergaming has only been tested in people with mild PD. To address these concerns, we recommend that large, robust randomised controlled trials be conducted in order to establish the safety and effectiveness of exergaming for rehabilitation in people with PD with a range of disease severity. In addition, to facilitate future reviews and meta-analysis of exergaming in PD we recommend i) future studies should report standard measures of disease severity (Hoehn and Yahr, and UPDRS III), medication status (levodopa equivalent daily dosage) and cognitive status (MMSE or MoCA); and ii) objective and subjective measures of safety, and the participants’ ability to play the games should be reported.

This review has also identified several important considerations when designing games for people with PD. To improve the effectiveness and adherence to exergaming interventions in people with PD, we recommend games designed for PD should: i) target specific clinical features of PD; ii) be easier than commercial games; iii) avoid negative feedback; iv) include very clear instructions and goals; iv) introduce cognitively demanding aspects slowly and sparingly; and v) examine the use of new exergaming systems that do not require balance platforms or handheld controller.

## Conclusion

Initial studies have shown that exergaming is a feasible intervention to improve motor symptoms in people with PD, however evidence is still lacking regarding its safety and clinical effectiveness. Exergaming may augment exercise therapy for people with PD although the use of commercial exergames may prove too complex for people with PD. Exergames specifically tailored towards PD symptoms may help improve both player enjoyment, motivation and effectiveness.

## Competing interests

The authors declare that they have no competing interests.

## Authors' contributions

GB carried out the initial search strategy and drafted the manuscript. BG was the second reviewer to carry out the quality assessment on the relevant articles with GB and helped to draft the manuscript. LR structured the review and helped to draft the manuscript. All authors read and approved the final manuscript.
